# Medical insurance benefits and health inequality: evidence from rural China

**DOI:** 10.3389/fpubh.2024.1363764

**Published:** 2024-05-22

**Authors:** Xueyi Wang, Cheng Qin

**Affiliations:** ^1^School of Economics, China-ASEAN Institute of Financial Cooperation, Guangxi University, Nanning, China; ^2^School of Business, Guangxi University, Nanning, China

**Keywords:** China, integrated medical insurance system, rural residents, health inequality, health

## Abstract

Alleviating health inequality among different income groups has become a significant policy goal in China to promote common prosperity. Based on the data from the China Health and Retirement Longitudinal Study (CHARLS) covering the period from 2013 to 2018, this study empirically examines the impact of Integrated Medical Insurance System (URRBMI) on the health and health inequality of older adult rural residents. The following conclusions are drawn: First, URRBMI have elevated the level of medical security, reduced the frailty index of rural residents, and improved the health status of rural residents. Second, China exhibits “pro-rich” health inequality, and URRBMI exacerbates health inequality among rural residents with different incomes. This result remains robust when replacing the frailty index with different health modules. Third, the analysis of influencing mechanisms indicates that the URRBMI exacerbate inequality in the utilization of medical services among rural residents, resulting in a phenomenon of “subsidizing the rich by the poor” and intensifying health inequality. Fourth, in terms of heterogeneity, URRBMI have significantly widened health inequality among the older adult and in regions with a higher proportion of multiple-tiered medical insurance schemes. Finally, it is suggested that China consider establishing a medical financing and benefit assurance system that is related to income and age and separately construct a unified public medical insurance system for the older adult population.

## 1 Introduction

The pursuit of equality is a key objective of healthcare systems, and worldwide consensus holds that everyone equally owns the right to health and equal health need ought to be treated equally regardless of income, identity, race or any other factors. However, individuals actually have different socioeconomic conditions, with high earners receiving better health education and enjoying better medical accessibility and so on, which bring better health outcome. Socioeconomic inequality in health indeed exists no matter in developed countries or developing countries. Health inequality mainly occurs among different income groups, especially in rural areas. In recent years, the income differentiation gap in rural areas of China has been widening. The low-income population is highly likely to fall into or even be trapped in the “health poverty” trap, where poor health leads to poverty, and poverty leads to worse health.

The medical insurance system is an important institution for alleviating the burden of medical expenses, promoting the utilization of medical services, improving the health of the population, enhancing the well-being of the people, and upholding social equity and justice. In 2003, the New Rural Cooperative Medical System (NCMS) targeting farmers was officially piloted. However, compared to the Urban Resident Medical Insurance System(URMI) possessed by urban residents, the medical insurance benefits for rural residents are significantly lower. In 2016, the State Council issued the “Opinions on Integrating the Basic Medical Insurance Systems for Urban and Rural Residents,” which mandated the integration of the URMI and the NCMS nationwide, aiming to establish a unified Urban–Rural Resident Basic Medical Insurance (URRBMI). The URRBMI has elevated the medical security level for rural residents. However, considering the large income disparities among rural residents, whether the improvement in medical insurance benefits promotes the health of low-income groups more than that of high-income groups is crucial in determining whether the medical insurance policy has met the requirements of fairness and justice.

Significant health social stratification exists in both developed and developing countries (1, 2). Some scholars have found that the greatest beneficiaries of medical insurance are those with lower income and poorer health conditions (3–5). However, there is still significant debate regarding whether medical insurance can effectively reduce health inequality. Some scholars argue that since the wealthy utilize a larger portion of the medical insurance fund, health insurance actually contributes more to improving the health of the affluent (6–12). Some scholars have already studied the impact of China’s medical insurance on health inequality. Xie (13) and Gu and Liu (14) found that medical insurance led to an increase in inequality, showing unequal health and medical service utilization between the rich and the poor. Zhou et al. (15) found that in urban resident medical insurance, the low-income population’s medical services and reimbursement amounts were significantly lower than those of the high-income population. However, some scholars’ research presents different perspectives. Studies by Ma and Zhao (16) discovered that although the health inequality among children from different income families in China was expanding, with children from higher-income families showing better health compared to those from lower-income families, the introduction of medical insurance significantly mitigated the widening of this health inequality. Additionally, Some scholars have already conducted research on the relationship between URRBMI and income inequality, but the results vary (17–20).

Existing literature does not elaborate in detail on the relationship between medical insurance and health inequality. This study, set against the backdrop of enhanced medical insurance for rural residents in China, utilizes data from the China Health and Retirement Longitudinal Study (CHARLS) from 2013 to 2018 to explore its impact on the health disparities among different income groups in rural areas. The marginal contribution of this paper lies in the use of the frailty index, which more accurately reflects the health status, focusing on the rural older adult population that is in poorer health and more significantly affected by medical insurance. This approach not only better reflects the policy impact but also develops a theoretical framework for medical insurance’s effect on health inequality, examining the corresponding impact mechanisms.

## 2 Theoretical background

**Figure 1:**
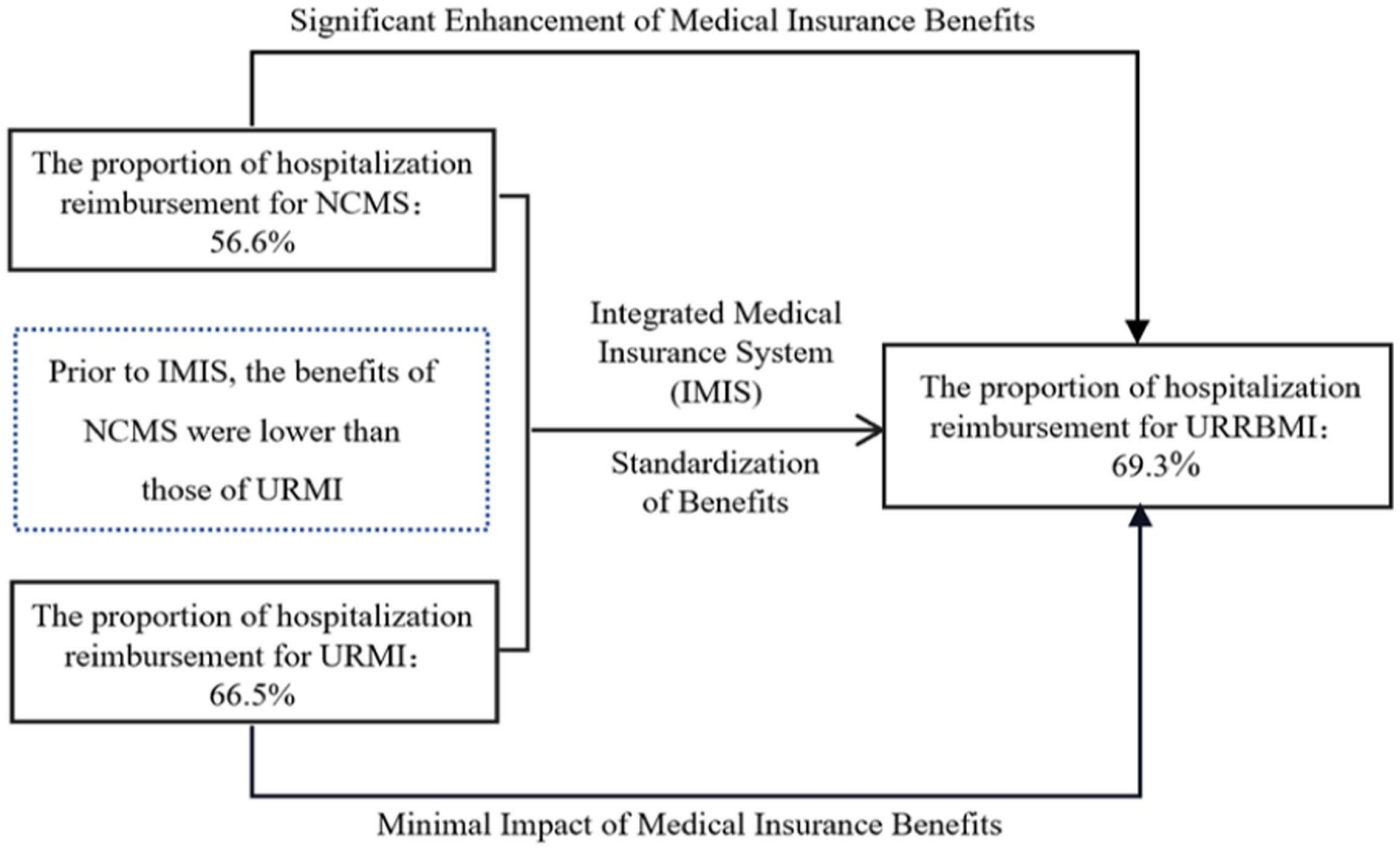
Diagram illustrating the URRBMI in China.

URRBMI has significantly improved the medical insurance benefits for rural residents (21, 22). Taking the most significant change, the hospitalization reimbursement ratio, as an example: before integration, the reimbursement ratio for hospitalization expenses within the coverage policy of the NCMS was approximately 56.6%, and for the URMI, it was about 66.5%. After URRBMI, the reimbursement ratio for hospitalization expenses within the coverage policy increased to approximately 69.3% (Figure 1). Rural residents are often engaged in agricultural or physically demanding labor, leading to greater health burdens. The demand for medical services is higher among the low-income groups within rural areas (23–27). From the perspective of medical insurance compensation and equity of benefits, whether the medical insurance system can reduce income-related health inequality depends on whether it can better promote medical services access and greater health improvement for low-income individuals.

On the one hand, enhancing the benefits of medical insurance is likely to increase the compensation for medical expenses among low-income individuals, thereby increasing their utilization of medical services. This is a crucial pathway to promote equity in health related to income. The key lies in the extent to which the existing medical insurance reimbursement can meet and stimulate the medical motivation of low-income individuals (28–31).

**Figure 2:**
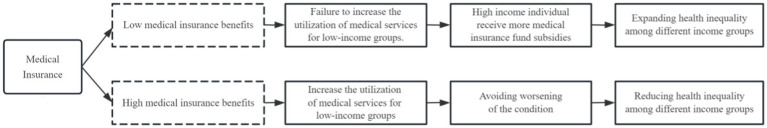
Analysis of the health impact of medical insurance on different income groups.

On the other hand, higher-income individuals often reside in economically developed regions where medical facilities are more advanced and concentrated. The basic medical insurance system establishes a deductible threshold, and due to economic constraints, low-income individuals might opt for cheaper medications that may not meet the reimbursement requirements of the deductible. The medical insurance system includes an upper limit for reimbursement, and even within the reimbursable range, there are often limitations on reimbursement percentages. The reimbursement ratio of basic medical insurance is not extremely high, and it only covers medications listed in the insurance catalog. Imported or high-value drugs and services might not be covered or may receive only partial reimbursement. Due to the constraints mentioned above, individuals with higher income, thanks to their greater purchasing power, might ultimately be more capable of accessing more medical services and receiving more insurance fund subsidies, thus exacerbating health inequality (6, 18, 20, 32) (Figure 2).

This study develops an economic model to analyze the impact of improved medical insurance coverage on the medical disparities among different income groups:

Assuming w represents income, M represents medical expenditures, and c represents other consumption. The price of other consumption is normalized to 1, while the price of medical expenditures is denoted as p. In this study, the utility function is modeled in a Cobb–Douglas form, specifically $u\left(c_{i},M_{i}\right)=c_{i}^{\alpha }M_{i}^{1-\alpha }$. Medical insurance, subsidized by a certain proportion, can be viewed as reducing the effective price of medical services. Since medical services is considered a normal good, an increase in income leads to an increase in demand. Let us assume the government provides a reimbursement rate of k for each household, satisfying the overall budget constraint:


(1) $$c_{i}+\left(1-k\right)pM_{i}=w_{i}$$

Under the budget constraint and utilizing the utility function $u\left(c_{i},M_{i}\right)=c_{i}^{\alpha }M_{i}^{1-\alpha }$, we can derive the optimal solution for medical expenditures:


(2) $$M_{i}^{1}=\frac{\left(1-\alpha \right)w_{i}}{\left(1-k\right)p}$$

In order to more accurately measure the benefits under the URRBMI, this study defines the reimbursement ratio of URRBMI as $k_{1}$, and the reimbursement ratio of the NCMS as $k_{2}$, where $k_{1}$ > $k_{2}$. The increase in medical resource utilization is denoted as *s_i_*. Assuming that household *i*’s optimal medical expenditures under the URRBMI are $M_{i}^{*}$, and the optimal medical expenditures under the original NCMS reimbursement level are $M_{i}^{0}$, then si can be calculated as:


(3) $$s_{i}=M_{i}^{*}-M_{i}^{0}$$

The optimal solution for the expansion of medical consumption is:


(4) $$s_{i}^{1}=\frac{\left(k_{1}-k_{2}\right)\left(1-\alpha \right)w_{i}}{\left(1-k_{1}\right)\left(1-k_{2}\right)p}$$

Taking the derivative of $s_{i}^{1}$ with respect to income $w_{i}$ yields:


(5) $$\frac{\partial s_{i}^{1}}{\partial w_{i}}=\frac{\left(k_{1}-k_{2}\right)\left(1-\alpha \right)}{\left(1-k_{1}\right)\left(1-k_{2}\right)p}>0$$

It can be observed that as income increases, the expansion of medical resource consumption also increases. In other words, higher-income individuals benefit more from the URRBMI, thus enhancing the health improvement effect for this group. Furthermore, this study conducts a static analysis by examining the changes in budget lines and indifference curves.

**Figure 3:**
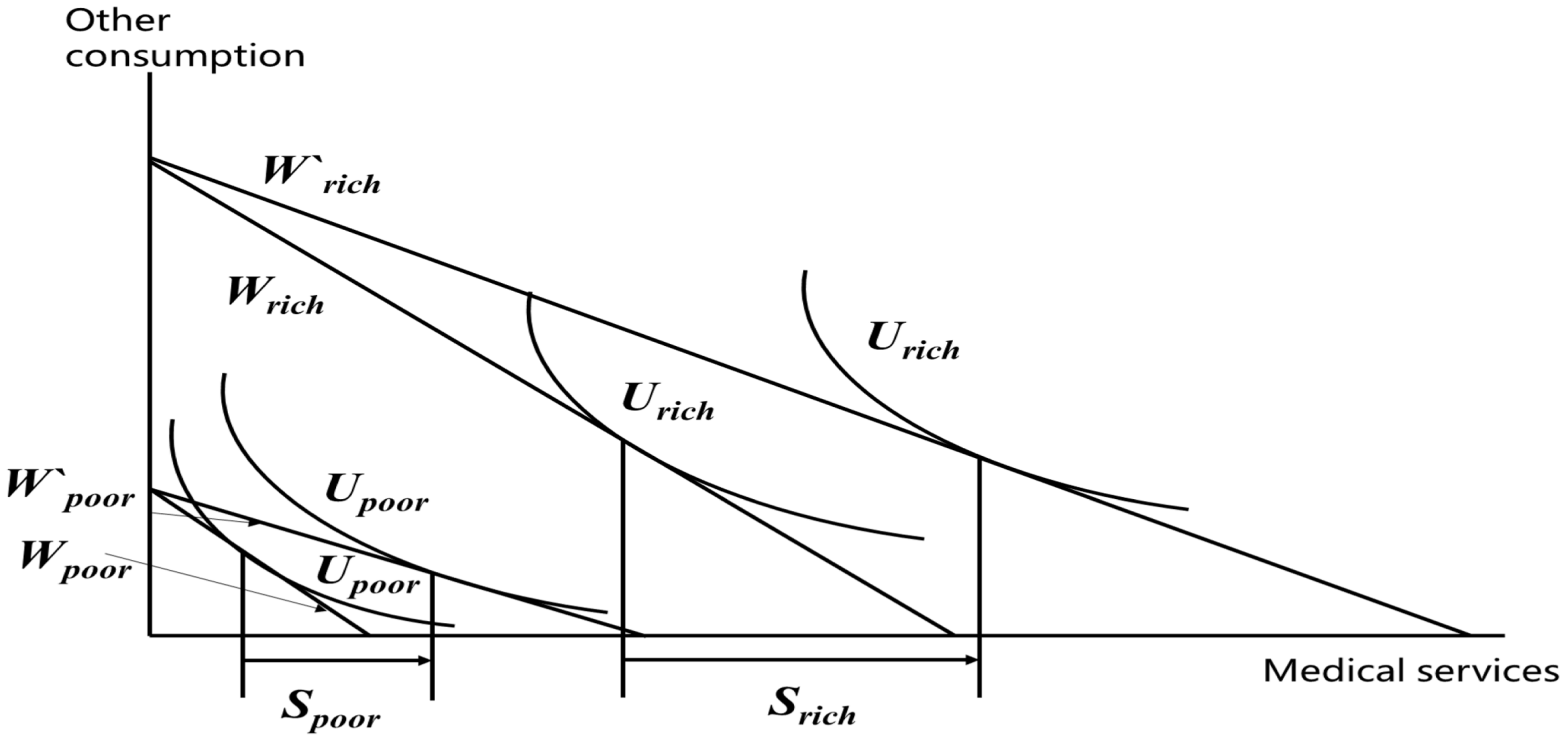
Static analysis of consumption for different income groups.

Figure 3 illustrates the consumption of medical services for low-income and high-income households. $w_{\text{poor}}$ and $w_{\text{poor}}^{,}$ represent the budget lines for low-income households participating in the NCMS and the URRBMI with increased reimbursement rates, respectively. $U_{\text{poor}}$ indicates the indifference curve for low-income households. Similarly, $w_{\text{rich}}$ and $w_{\text{rich}}^{,}$ denote the budget lines for high-income households participating in NCMS and the URRBMI after increased reimbursement rates, respectively. $U_{\text{rich}}$ represents the indifference curve for high-income households. The increased medical consumption for low-income households in the URRBMI is represented as $s_{\text{poor}}$, while the increased consumption for high-income households is denoted as $s_{\text{rich}}$. It’s evident that $s_{\text{rich}}>s_{\text{poor}}$, indicating that in the URRBMI, high-income households experience a larger increase in medical service consumption compared to low-income households, implying greater benefits for high-income households from the policy.

Furthermore, the design of the medical insurance system also favors higher-income individuals. On one hand, medical services in China are primarily concentrated in public medical institutions and economically developed areas, where rural residents also tend to have relatively higher incomes, resulting in better accessibility to high-quality medical resources. On the other hand, after the URRBMI, the administration shifts from county-level coordination to city-level coordination. However, rural residents seeking medical treatment outside their own city still fall under the category of “out-of-town treatment,” with lower reimbursement rates and the requirement for prior reporting to be eligible for reimbursement. This significantly limits the medical services access of low-income individuals in underdeveloped regions. In contrast, high-income individuals, due to their higher health awareness and motivation to seek care at higher-level medical institutions, are able to access better medical services and receive greater compensation, thereby significantly improving their health status.

Based on the theoretical analysis provided above, propose the following assumptions:

The URRBMI has widened the health inequality among different income groups within rural areas.

## 3 Methods

### 3.1 Data source

This study utilizes data from the China Health and Retirement Longitudinal Study (CHARLS), which targets individuals aged 45 and above in China. The survey employs a multi-stage stratified sampling method and has conducted four nationwide follow-up surveys in 2011, 2013, 2015, and 2018. Due to a limited number of samples participating in the URRBMI in 2011, this study excluded that year’s data and utilized data from the years 2013, 2015, and 2018.

Considering the focus of this study on analyzing the impact of the URRBMI on health inequality related to income among rural middle-aged and older adult individuals, rural samples were retained based on their household registration status and place of residence. To ensure a more accurate analysis of the policy effects of the URRBMI, this study excluded individuals who participated in URMI, those who were enrolled in multiple types of medical insurance, participants of commercial medical insurance, individuals who were not locally insured, and those who were not covered by any medical insurance. Only individuals participating in the NCMS or the URRBMI were included, while individuals with missing data in relevant variables were also excluded. As a result, a panel dataset with three waves was constructed, consisting of an effective sample size of 15,899 individuals.

### 3.2 Variables

#### 3.2.1 Health

*Health* indicators use the frailty index, with values ranging from 0 to 1. A higher value indicates poorer health. The frailty index includes the following 6 modules:

(1) Self-rated Health(SH): How do you perceive your health status? Ratings “very good,” “good,” “fair,” “bad,” and “very bad” are defined as 0.2, 0.4, 0.6, 0.8, and 1, respectively.
(2) Instrumental Activities of Daily Living (IADL): Do you have difficulties in “managing money, shopping, cooking, making phone calls, and cleaning”? Difficulties are defined as 1; otherwise, it’s 0.
(3) Activities of Daily Living (ADL): Do you have difficulties in “bathing, getting up, using the toilet, eating, dressing, and making decisions”? Difficulties are defined as 1; otherwise, it’s 0.
(4) Functional Limitations (FL): Do you have difficulties in activities like “walking 100 meters, climbing stairs, reaching upward, getting up from a chair, bending over or kneeling, picking up a coin, lifting 10 kilograms”? Difficulties are defined as 1; otherwise, it’s 0.
(5) Mini-Mental State Examination (MMSE): Can you clearly answer questions about “the current year, month, date, season, day of the week, your memory condition, drawing a picture, depression level”? Similar to self-rated health, responses “very good,” “good,” “fair,” “bad,” and “very bad” are defined as 0.2, 0.4, 0.6, 0.8, and 1 respectively; other accurate responses are defined as 0, otherwise it’s 1.
(6) Chronic Disease (CD): Based on whether you have conditions like hypertension, hyperlipidemia, hyperglycemia, malignant tumors, chronic lung diseases, liver diseases, heart diseases, strokes, kidney diseases, gastrointestinal diseases, emotional and mental issues, memory-related diseases, rheumatism, and asthma. These are defined as 0 (absent) or 1 (present).

These 6 modules involve a total of 41 health variables, with specific formulas as follows:


(6) $$FI=\frac{\sum_{k=1}^{n}d_{i}}{n}$$

In the above equation, FI represents the frailty index, *n* = 41, and $d_{i}$=1 indicates that the *i*-th health variable is in a health deficit state, otherwise $d_{i}$=0.

#### 3.2.2 URRBMI

Based on the survey of individual participation status in CHARLS, if an individual is enrolled in the URRBMI, the variable takes the value of 1; if the individual is enrolled in the NCMS, the variable takes the value of 0.

#### 3.2.3 Control variables

Regarding control variables, this study selected individual demographic and household characteristics. The specific definitions of variables and descriptive statistics are shown in Table 1.

**Table 1:** Descriptive statistics of the variables.

Variable name	Variable definition	Mean	Standard deviation
FI	/	0.1747	0.1131
URRBMI	URRBIM =1, NCMS =0	0.0692	0.2538
Age	/	61.1720	9.4902
Education years^*^	/	3.9186	4.2041
Marital status	1 = Married, 0 = Unmarried	0.8168	0.3868
Gender	1 = Female, 0 = Male	0.5360	0.4987
*Per Capita* income	Logarithm of *Per Capita* Annual Income	6.8030	2.9949
Smoking	1 = Yes, 0 = No	0.2490	0.4324
Drinking	1 = Yes, 0 = No	0.3287	0.4698
Exercise	1 = Yes, 0 = No	0.9302	0.2548
Number of children	Number of biological and step children	2.7135	1.5311
Household Cleanliness Level	1 = Very Clean, 2 = Quite Clean, 3 = Clean, 4 = Average, 5 = Not clean	3.1613	1.1518
Toilet type	1 = Squatting toilet, 0 = Sitting toilet	0.8691	0.3373
Tap water	1 = Yes, 0 = No	0.6659	0.4716

*: No education or incomplete primary school = 0; Primary school = 6; Junior high school = 9; Senior high school or vocational school = 12; Junior college = 15; Bachelor’s degree = 16; Master’s degree = 19; Doctorate = 23.

### 3.3 Methodology and empirical strategy

#### 3.3.1 The health concentration index

The health concentration index (HCI) is a commonly used measure to assess the degree of health inequality among different income groups (6, 18). To calculate the HCI, two elements must be included: first, a measure of health, which is usually a binary or continuous variable and cannot be a multi-ordered variable. In this study, the *Frailty Index* is used as the health measurement indicator. Second, a measure of economic status, specifically an indicator of income status. In this study, household *Per Capita Income* is used. The *HCI* is represented by the concentration curve of health (with the horizontal axis being the cumulative percentage of individuals sorted by income from low to high, and the vertical axis being the cumulative percentage of individuals sorted by health status) and twice the area between the concentration curve and the equity line (the 45-degree diagonal line). Therefore, this study follows the approach of Wagstaff et al. (33) and defines the expression for the *HCI* as follows:


(7) $$HCI=\frac{2}{H}\mathrm{cov}\left(XR_{i},R_{i}\right)=\frac{2}{nH}\sum XR_{i}R_{i}-1$$

The *HCI* takes values between −1 and 1. H represents the average health status of the sample. $R_{i}$ denotes the rank of the *i*th individual in the sample when individuals are ranked by their income from low to high. This rank is calculated as (i)/n. Since the frailty index used in this study is a negative indicator where higher values imply worse health, a negative value of the *HCI* that the health status is better among higher income individuals, suggesting a tendency towards health inequality favoring the higher income group. Conversely, a positive value of the *HCI* indicates that the health status is better among lower income individuals, indicating a tendency towards health inequality favoring the lower income group.

#### 3.3.2 The decomposition of HCI

The HCI measures the extent of income-related health inequality. However, what we are more concerned about is identifying the factors that influence the degree of income-related health inequality. Therefore, the next step is to decompose HCI. Following the approach of Peng et al. (6) and Fan et al. (18), the HCI is decomposed into contributions from various health factors. The contribution of each factor can be divided into its direct impact on health (measured by elasticity) and its indirect impact on income-related health inequality by covering different income groups (measured by the Concentration Index of that factor).

Hence, it is necessary to calculate the Concentration Index (CI) and elasticity of each influencing factor. First, we need to analyze the marginal effects of various factors on health. This study adopts a Fixed Effects Model (FE) for identification, and the specific model setup is as follows:


(8) $$FI_{\text{it}}=\alpha_{0}+\alpha_{1}\text{insuranc}e_{\text{it}}+\alpha_{2}X_{\text{it}}+\varepsilon_{\text{it}}$$

$FI_{\text{it}}$ represents the frailty index of individual *i* at time *t*, and $\text{insuranc}e_{\text{it}}$ indicates whether individual *i* joined the URRBMI in period *t*. $X_{\text{it}}$ represents the demographic and household characteristics of individual *i* in period *t*, and $\varepsilon_{\text{it}}$ is the random error term.

Subsequently, based on Eq. 8, both sides are simultaneously calculated for the *CI*. The corresponding elasticity is derived from the means of each factor. Then, the *CI* of each factor is calculated, followed by a weighted average using elasticity as weights. This achieves the decomposition of HCI, as shown in the equation:


(9) $$HCI=\sum \eta_{k}CI_{k}+\frac{CG_{k}}{\mu }$$

HCI stands for the Health Concentration Index. $CI_{k}$ represents the Concentration Index for factor *k*. $\eta_{k}(\eta_{k}=\beta_{k}{\bar{x}}_{k}/\mu$) signifies the elasticity of health demand for factor *k*. $\beta_{k}$ stands for the regression coefficient of factor *k*. ${\bar{x}}_{k}$ and μ correspond to the means of factor *k* and the frailty index, respectively. The elasticity of demand $\eta_{k}$ illustrates the impact of variations in factor *k* on the frailty index. $CG_{k}/\mu$ denotes the influence of the disturbance term on the HCI.

#### 3.3.3 Decomposition of HCI variation

Due to the utilization of a three-wave panel dataset in this study, the variation in the HCI can also be analyzed from a dynamic perspective. Following the approach of Peng et al. (6) and Fan et al. (18), the Oaxaca decomposition is employed in this study to break down the changes in the Health Concentration Index across different periods into variations in the concentration indices of various factors and changes in the elasticity of demand. The specific decomposition formula is as follows:


(10) $$\begin{array}{l} \Delta CI=\sum \eta_{kt-1}\left({CI}_{kt}-{CI}_{kt-1}\right)+\sum {CI}_{kt}\left(\eta_{kt}-\eta_{kt-1}\right)\\ \;+\Delta \left(\frac{{CG}_{k}}{\mu }\right) \end{array}$$

ΔCI represents the HCI variation, $CI_{kt}$ and $CI_{kt-1}$ are the CI of various factors in periods *t* and *t−1*, and $\eta_{kt}$ and $\eta_{kt-1}$ are the demand elasticities of various factors in periods *t* and *t−1*, respectively.

## 4 Results

### 4.1 Baseline regression

**Figure 4:**
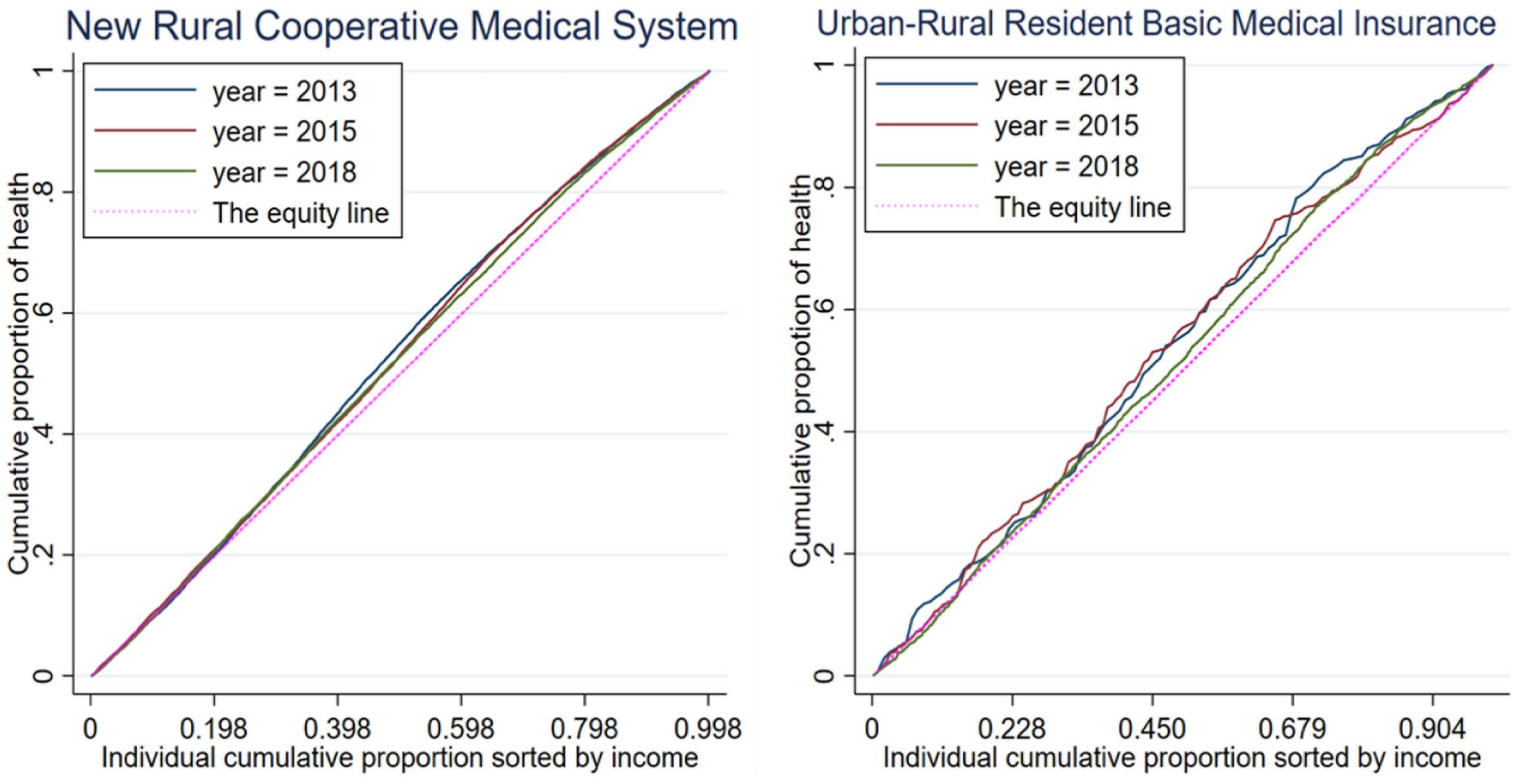
Health concentration index (HCI) curve.

Firstly, the research sample is divided into two groups: those covered by the NCMS and those covered by the URRBMI. Based on the aforementioned research methodology, the study has constructed health concentration curves for different years (Figure 4). The health indicator used in this study is the frailty index, with higher values indicating worse health conditions. The health concentration curves are positioned above the equity line (the 45-degree diagonal line), indicating that individuals with higher incomes have better health conditions.

From Figure 4, it can be observed that for both the NCMS-covered sample and the URRBMI-covered sample, their respective health concentration curves are situated above the equity line. This implies that in rural areas of China, irrespective of whether individuals have undergone the URRBMI, there exists health inequality related to income. Furthermore, it is evident that the health concentration curve of the URRBMI-covered sample deviates more significantly from the equity line, suggesting that individuals covered by URRBMI experience worse income-related health inequality. The URRBMI indeed leads to an expansion of health inequality associated with income, benefiting individuals with higher incomes in terms of health status.

However, this study also uncovers a trend of decreasing income-related health inequality over time for both the NCMS and URRBMI-covered groups. The study also calculates health concentration indices for different insured samples in different years, as well as for the combined years, as presented in Table 2.

**Table 2:** Health concentration index (HCI) analysis.

Year	All samples	URRBIM	NCMS
2013	−0.0568	−0.0788	−0.0564
2015	−0.0508	−0.0767	−0.0499
2018	−0.0416	−0.0419	−0.0403
All years	−0.0320	−0.0351	−0.0310
N	15,899	1,102	14,797

The overall HCI for middle-aged and older adult individuals in rural areas of China is −0.0320 (Table 3), indicating that individuals with higher income levels tend to have better health. This study employs a FE model to decompose the HCI based on regression coefficients, calculating the elasticity of various variables. The concentration indices for each variable are then calculated, and the contribution rates of each variable to income-related health inequality are computed (by dividing the total contribution of each variable by the HCI).

**Table 3:** Decomposition of income-related health inequality among rural middle-aged and older adult individuals.

Variable	Coefficients	Elasticity	CI	Contribution rate (%)
URRBMI	−0.0126	−0.0050	0.1697	2.65
Age	0.0009	0.3252	−0.0059	6.04
Education years	−0.0010	−0.0226	0.0517	3.70
Marital status	0.0064	0.0300	−0.0040	0.37
*Per Capita* income	−0.0004	−0.0153	0.2308	11.22
Smoking	0.0268	−0.0383	0.0519	6.20
Gender	0.0170	0.0517	−0.0491	8.08
Exercise	−0.0205	−0.1092	0.0012	0.42
Drinking	0.0060	−0.0112	0.0583	2.05
Number of children	0.0012	0.0132	−0.0137	0.27
Household cleanliness level	0.0024	0.0434	−0.0025	0.34
Toilet type	−0.0056	−0.0277	−0.0154	−1.33
Tap Water	−0.0050	−0.0193	0.0190	1.15

As shown in Table 3, the contribution rate of the URRBMI is 2.65%. A positive (negative) contribution rate indicates that the variable exacerbates (mitigates) health inequality among individuals with different income levels. The positive CI of the URRBMI indicates that it covers a larger proportion of high-income individuals, while the elasticity suggests that health status is significantly affected by the implementation of URRBMI. The combined effect of both factors makes the URRBMI an important contributor to health inequality among middle-aged and older adult individuals with different income levels in rural China. Among other control variables, this study also found that factors such as Education Years, *Per Capita* Income, smoking, and Drinking are important contributors to income-related health inequality. For instance, the contribution rate of *Per Capita* Income is as high as 11.22%. As income distribution becomes more unequal, higher-income individuals can access better healthcare coverage, leading to greater improvements in health and thereby widening the gap in health inequality among individuals with different income levels.

During the early stages of the NCMS development, Wagstaff (34) and Lei et al. (35) already identified the phenomenon of subsidies from the poor to the rich within the NCMS. The findings of this study are consistent with the conclusions of Fan (18) and He et al. (20), indicating that the URRBMI widens the health inequality among different income groups. This study posits that China’s administratively-based medical insurance system allows higher-income individuals to enjoy more medical services and greater medical fund subsidies, thereby providing them with better health coverage. This observation aligns with previous research, as higher-income individuals possess greater payment capacity, enabling them to access better medical services and receive higher medical insurance reimbursements (33, 36).

On one hand, due to higher income levels among residents in economically developed regions, medical services in China are primarily concentrated in public medical institutions. These institutions have higher management levels, receive substantial financial subsidies, and possess more centralized medical resources. Consequently, in rapidly developing economic areas, medical resources, particularly high-quality ones, experience significant improvement. Higher-income individuals have better access to these improved medical resources, and the enhancement of medical insurance benefits has a stronger motivating effect on their medical-seeking behavior.

On the other hand, URRBMI also has certain regional limitations. Seeking medical treatment outside one’s own city or locality is categorized as cross-regional medical treatment, with reimbursement rates being lower compared to local treatment. Moreover, individuals seeking cross-regional treatment need to report and gain approval for reimbursement. This significantly restricts the medical demand of low-income individuals in less developed regions. In contrast, higher-income individuals, possessing a higher level of medical awareness and stronger motivation to seek medical treatment at advanced medical institutions, can access better medical services and receive more compensation. Consequently, their overall health status improves significantly. Therefore, it can be said that increasing medical insurance benefits itself may not lead to health inequality among different income groups; rather, the inequality is partially caused by the inadequate design of the system, resulting in health disparities related to income.

### 4.2 The trend and decomposition of health inequality

As shown in Table 4, in the years 2013, 2015, and 2018, the contribution rates of the URRBMI to the HCI were 0.07, 0.06, and 3.03%, respectively. After the large-scale URRBMI nationwide in 2016, the contribution rate of URRBMI to the HCI sharply increased from a very slight level to 3.03%. The reason lies in the fact that with the widespread implementation of URRBMI across the China, the health improvement effects on the high-income population were more pronounced, leading to an increase in health inequality among rural individuals with different income levels. In 2013, URRBMI had a negative impact on health, which might be attributed to the initial implementation of the insurance leading to improved coverage, potentially resulting in excessive medical treatment such as antibiotic misuse. Thus, the enhancement of medical insurance benefits might have caused certain harm to health. However, individuals with lower incomes may lack adequate healthcare knowledge and face certain negative consequences during medical processes, contributing to the widening health inequality across different income groups.

**Table 4:** Annual contributions of URRBMI to HCI.

	Coefficients	HCI	Elasticity	Contributions	Contribution rate (%)
2013	0.0031	−0.0943	0.00039	−0.000037	0.07
2015	−0.0028	0.0605	−0.00046	−0.000028	0.06
2018	−0.0195	0.0814	−0.0155	−0.001261	3.03

This study decomposes the variation in HCI, dividing them into three intervals: 2013–2018, 2013–2015, and 2015–2018. The decomposition results indicate that the contribution of URRBMI to the variation in HCI during 2013–2018 is mainly due to elasticity changes (Table 5). The impact of URRBMI on the HCI is not significant, as the integration covers all rural residents, with little variation in coverage among different income groups. The variation in HCI is more likely a result of the impact of joining the URRBMI on health rather than the coverage of the integration itself. This may be attributed to the fact that the effect of URRBMI on health improvement is mainly observed among high-income individuals.

**Table 5:** Sources of contribution of URRBMI to changes in HCI.

Interval	Total contribution	Contribution to HCI	Contribution to elasticity
2013–2018	−0.001224	0.0000693	−0.001293
2013–2015	0.0000092	0.0000611	−0.0000519
2015–2018	−0.001231	−0.0000096	−0.001223

### 4.3 Path analysis

Based on theoretical analysis, medical services utilization is one of the most crucial pathways affecting health. The expansion of URRBMI has widened the health inequality associated with income, largely due to the fact that it has predominantly facilitated medical services utilization among higher-income individuals. Consequently, this study investigates the impact of URRBMI on medical services utilization inequality among rural middle-aged and older adult individuals.

In the field of health economics, medical services utilization inequality can be categorized into vertical equity and horizontal equity. Vertical equity refers to the unequal treatment of individuals with different medical needs, while horizontal equity pertains to equal treatment for equal medical needs. Health economics generally assumes that vertical equity has been achieved (6). Therefore, following the design of studies by Fan et al. (18) and Peng et al. (6), this research defines medical services utilization equity as horizontal equity. In this context, an individual’s medical services utilization should be determined by factors related to their health condition, age, gender, and other similar needs-based variables, rather than being influenced by non-needs-based variables like occupation, income, and social status (as is the case in this study with factors such as URRBMI and personal and family characteristics like income). If medical services utilization is influenced by non-needs-based variables, it implies the presence of horizontal inequity in medical services utilization.


(11) $$y_{i}=\alpha +\sum_{k}\gamma_{k}x_{ki}+\sum_{p}\delta_{p}z_{\text{pi}}+\varepsilon_{i}$$

$y_{i}$ represents individual medical service utilization, where $x_{k}$ represents k “need-based” variables, including age, gender, and physical health status. $z_{p}$ represents *p* “non-need-based” variables. By multiplying the actual values of “need-based” variables and the average values of “non-need-based” variables by the corresponding regression coefficients from Eq. 11, one can obtain the individual’s expected medical service utilization based on their need-based characteristics.

This study divided income into high-income and low-income groups based on the median income. The results indicate that, whether in outpatient or inpatient settings, high-income individuals have a better difference between actual and expected medical service utilization compared to low-income individuals. As shown in Table 6, in terms of outpatient probability, the actual utilization for the low-income group is 0.0066 lower than the expected utilization, while for the high-income group, the actual utilization is 0.0100 higher than expected. Regarding outpatient frequency, the actual utilization for the low-income group is 0.0314 lower than the expected, while for the high-income group, the actual utilization is 0.0105 higher than expected. In terms of inpatient probability, the actual utilization for the low-income group is 0.0223 lower than the expected, while for the high-income group, the actual utilization is 0.0325 higher than expected. Concerning inpatient frequency, the actual utilization for the low-income group is 0.0123 lower than the expected, while for the high-income group, the actual utilization is 0.0240 higher than expected. Therefore, it can be concluded that among China’s rural older adult population, high-income individuals indeed receive more medical services compared to low-income individuals, indicating the presence of income-related medical service utilization inequality.

**Table 6:** Differences in medical service utilization among different income groups.

	Probability of outpatient visits in the past month			Number of outpatient visits in the past month		
Horizontal inequity index	0.008			0.0122		
Group	Actual utilization	Expected utilization	Difference	Actual utilization	Expected utilization	Difference
Low-income	0.1993	0.2059	−0.0066	0.4349	0.4663	−0.0314
High-income	0.204	0.194	0.01	0.4556	0.445	0.0105

	Probability of hospitalization in the past year			Number of hospitalization in the past year		
Horizontal inequity index	0.0148			0.0146		
Group	Actual utilization	Expected utilization	Difference	Actual utilization	Expected utilization	Difference
Low-income	0.1448	0.1671	−0.0223	0.2231	0.2354	−0.0123
High-income	0.1557	0.1232	0.0325	0.245	0.2211	0.024

Furthermore, following the approach of Jie (2009), Peng et al. (6), and Fan et al. (18), this study measured the horizontal inequality index. By distinguishing between need-based and non-need-based variables, the aim was to estimate the remaining inequality after accounting for differences in medical service utilization of need-based. This was achieved by subtracting the expected concentration index of medical utilization from the actual concentration index of medical utilization. As shown in Tables 4–6, the horizontal inequality indices for outpatient probability, outpatient frequency, inpatient probability, and inpatient frequency were 0.0080, 0.0122, 0.0148, and 0.0146, respectively. This again confirms the existence of medical service utilization inequality in rural China.

In order to analyze the contributions of variables such as the URRBMI to the inequality in medical service utilization, this study decomposed the concentration index of medical service utilization. As shown in Table 7, the variables were divided into need-based and non-need-based categories. It is evident that the medical service utilization among rural older adult individuals is influenced not only by need-based variables but also by non-need-based variables. The contributions of URRBMI to the inequality in outpatient probability, outpatient frequency, inpatient probability, and inpatient frequency related to income were 4.17, 1.22, 1.46, and 0.33%, respectively. Path analysis confirmed that, compared to the NCMS, the URRBMI did indeed lead to greater utilization of medical services by high-income rural older adult individuals, thus exacerbating income-related medical inequality and increasing the disparity in health outcomes among different income groups.

**Table 7:** Decomposition of income-related inequality in medical service utilization.

	Outpatient probability	Frequency	Inpatient probability	Inpatient frequency
Contribution rates of need-based variables				
Age	40.35%	18.59%	26.78%	21.74%
Gender	59.80%	65.79%	−41.52%	−25.61%
Health Status	−26.14%	−14.33%	41.05%	37.30%
Contribution rates of non-need-based variables				
URRBMI	4.17%	1.22%	1.46%	0.33%
*Per Capita* Income	38.43%	30.16%	31.73%	28.14%

### 4.4 Heterogeneity analysis

The sample population of this study comprises rural middle-aged and older adult individuals. With increasing age, there is a decline in individual health levels, leading to significant health disparities among middle-aged and older adult individuals. Furthermore, in recent years, income inequality within rural areas has also been growing. Therefore, this study further investigates the effect of URRBMI on the income-related health inequality among rural middle-aged and older adult populations.

The middle-aged group consists of individuals aged 45 to 60, while the older adult group includes those aged 60 and above. As shown in Table 8, the contribution of URRBMI to the income-related health inequality among rural middle-aged and older adult individuals is 2.36 and 6.32%, respectively. The difference between the two is as large as 3.96 percentage points. URRBMI significantly exacerbates the income-related health inequality among rural older adult individuals compared to middle-aged individuals. This could be attributed to the poorer health status of the older adult, higher healthcare consumption needs, and the fact that medical insurance integration increases medical consumption for higher-income individuals, leading to health improvements primarily among this group. This phenomenon is more pronounced among the older adult population due to their greater medical needs and vulnerability.

**Table 8:** Age and tier heterogeneity analysis of the impact of URRBMI on HCI.

	Middle-aged group	Older adult group	Single-tier	Multi-tier
Mean of Health	0.1401	0.2008	0.1761	0.1633
HCI	−0.0424	−0.0158	−0.0298	−0.0497
Coefficient of URRBMI	−0.0124	−0.0172	−0.0118	−0.0182
Mean of URRBMI	0.0516	0.0812	0.0642	0.1085
Elasticity of URRBMI	−0.0064	−0.0049	−0.0043	−0.0121
Concentration index of URRBMI	0.1556	0.2025	0.1583	0.2125
Contribution rate of URRBMI (%)	2.36%	6.32%	2.29%	5.17%
*N*	6,859	9,040	14,130	1769

Furthermore, after URRBMI, most cities offer only one level of medical insurance coverage. However, some cities have established different tiers of medical insurance coverage based on varying payment amounts to meet the diverse medical needs of different populations. Urban and rural residents can obtain different levels of medical insurance coverage based on their payment amounts. Relatively speaking, individuals with lower incomes tend to opt for lower payments and thus lower levels of medical coverage. However, when faced with health issues, lower-tier medical insurance coverage may struggle to provide access to high-quality medical services and more substantial medical insurance fund compensation. The different tiers of medical insurance coverage could potentially further exacerbate income-related health inequality.

As shown in Table 8, in regions with both a single-tier and multi-tier medical insurance system, the contribution of URRBMI to health inequality is 2.29 and 5.17%, respectively, showing a difference of nearly 1-fold. Therefore, while the policy practice of offering different tiers of medical insurance coverage based on varying payments can address the medical needs of diverse population groups, it can also amplify income-related health inequality. This is why related policy documents call for a gradual transition from a multi-tier to a single-tier medical insurance system over the course of 2 to 3 years.

### 4.5 Robustness test

This study further examines the robustness of the conclusions by changing the measurement indicators of individual health. As the calculation process of the health concentration index requires health indicators to be binary or continuous variables and not polytomous ordinal variables, adjustments are made to ensure the suitability of the health indicators.

In the self-rated health (SH) module, SH is categorized as 0 when reported as “very good” or “good,” and as 1 when reported as “fair,” “bad,” or “very bad.” In the Activities of Daily Living (ADL) module, difficulties in aspects such as “bathing, getting up, using the toilet, eating, dressing, and making decisions” are assigned a value of 1, while the absence of difficulties receives a value of 0. Similarly, in the Instrumental Activities of Daily Living (IADL) module, difficulties in aspects like “managing money, taking medications, shopping, cooking, making phone calls, and cleaning” are assigned a value of 1, with no difficulties assigned a value of 0. In the Chronic Diseases (CD) module, the presence of conditions such as hypertension, hyperlipidemia, hyperglycemia, malignancies, chronic lung diseases, liver diseases, heart diseases, strokes, kidney diseases, stomach diseases, emotional and mental issues, memory-related diseases, rheumatism, asthma, or any combination thereof, is given a value of 1, while the absence of these conditions receives a value of 0. In the Physical Function Limitations (FL) module, difficulties in aspects like “walking 100 meters, climbing stairs, reaching upward, standing up from a chair, bending or kneeling or squatting, picking up a coin, lifting 10 kilograms of weight” are assigned a value of 1, while the absence of these difficulties receives a value of 0. The Mini-Mental State Examination (MMSE), being originally a continuous variable, requires no adjustment.

From the robustness results, it can be observed that regardless of the replacement of health indicators, the URRBMI widens the health inequality among different income groups and particularly enhances the health of higher-income individuals. Among these indicators, the impact on health inequality is more pronounced in HS, CD, and MMSE, while it is relatively smaller for ADL, IADL, and FL. This could be attributed to the fact that SH, MMSE, and chronic diseases are more susceptible to the influence of medical insurance coverage. On the other hand, ADL, IADL, and FL are indicators of poorer health status, indicating a greater likelihood of disability. Improving medical insurance coverage may have a limited effect on improving health inequality among individuals with worse health and potential disabilities (Table 9).

**Table 9:** Robustness analysis of the impact of urban and rural residents’ medical insurance integration on health concentration index.

	SH	ADL	IADL	CD	FL	MMSE
HS	−0.0198	−0.1028	−0.1211	−0.0609	−0.0349	−0.0344
Coefficient of URRBMI	−0.0367	−0.0266	−0.0766	−0.0637	−0.0560	−0.1666
Mean of URRBMI	0.0820	0.0815	0.0815	0.0815	0.0823	0.0906
Elasticity of URRBMI	−0.0038	−0.0106	−0.0112	−0.0088	−0.0040	−0.0063
Concentration index of URRBMI	0.1934	0.1991	0.1992	0.1991	0.1860	0.1894
Contribution rate of URRBMI (%)	3.77%	2.05%	1.85%	2.89%	1.92%	3.46%
*N*	15,899	15,899	15,899	15,899	15,899	15,899

## 5 Discussion

This study demonstrates that high-income groups utilize medical services more frequently than low-income groups, resulting in greater improvements in health status among the former, thereby exacerbating health inequalities. Hence, merely enhancing medical insurance benefits will disproportionately benefit high-income individuals. It is imperative for future Chinese government policies on medical insurance to favor low-income individuals, allowing them to derive greater benefits from medical insurance.

The impact of the Urban and Rural Residents Basic Medical Insurance (URRBMI) on health inequalities among the older adult is notably pronounced. Currently, rural China is facing a severe aging issue, and medical insurance policies have a significant impact on the older adult, likely further widening health disparities among this demographic. This could destabilize rural China. The Chinese government should provide more effective medical welfare policies for rural older adult individuals, ensuring that every rural elder has access to ample medical insurance benefits, thus reducing social welfare inequalities.

China’s medical insurance system continues to maintain two parallel insurance schemes: the Urban Employee Medical Insurance and URRBMI, based on a dualistic occupational foundation. This system presents significant disparities in benefits and subsidies. Drawing on the experience from the previous integration of the New Rural Cooperative Medical Scheme and the Urban Residents Medical Insurance, special attention should be paid to the issue of health inequality expansion among different income groups when merging the Urban Employee Medical Insurance and URRBMI.

## 6 Limitation

This research has three major limitations: Firstly, it primarily focuses on the middle-aged and older adult populations, yet the utilization of medical services and health needs significantly differ across various stages of life, such as in children and young adults. As data continues to be enriched, further analysis will be conducted on the impact of medical insurance and enhanced medical insurance benefits on populations of different age groups, utilizing the continually evolving micro-databases. Secondly, due to limitations imposed by the indicators of the selected micro-databases, this study’s investigation into the pathways through which medical insurance affects health inequalities is not comprehensive. The research mainly considered the pathway of unequal access to medical services. Future studies will leverage the expanding micro-databases to explore a more diverse range of impact pathways, such as how medical insurance changes food consumption patterns, thereby affecting health inequalities. Thirdly, the sample size of research on the New Rural Cooperative Medical Scheme and urban and rural resident medical insurance is not balanced. This may to some extent underestimate the level of health inequality in Urban–Rural Resident Basic Medical Insurance (URRBMI), which means that the level of health inequality in urban and rural resident medical insurance may be higher than expected in this paper.

## 7 Conclusion

This study is based on the CHARLS data from 2013, 2015, and 2018. Using the HCI and its decomposition method, it empirically analyzes the impact of the URRBMI on the internal health inequality within rural areas. In China, the health level of rural middle-aged and older adult individuals exhibits inequality favoring those with higher incomes. The URRBMI exacerbates health inequality among different income groups, contributing to a rate of 2.65%.

Following the large-scale URRBMI in 2016, its contribution to the HCI substantially increased from a minimal level to 3.02%. Secondly, the contribution of the integration primarily stems from the elasticity changes. There is not a significant difference in coverage rates among different income groups; rather, the impact on HCI is due to the effect of implementing the URRBMI on health. This effect is likely to be more prominent among higher income individuals.

Thirdly, in terms of the pathway analysis, the URRBMI leads to greater usage of medical services by high-income rural older adult individuals, thereby amplifying the income-related medical inequality and ultimately intensifying health inequality related to income.

Lastly, in the context of heterogeneity analysis, the URRBMI has a more pronounced impact on health inequality related to income among rural middle-aged and older adult individuals and is more substantial for the older adult population and in areas with multiple-tiered medical insurance plans.

## Data availability statement

Publicly available datasets were analyzed in this study. This data can be found at: http://charls.pku.edu.cn.

## Author contributions

XW: Writing – review & editing. CQ: Writing – original draft, Writing – review & editing.
